# Identification of candidate repurposable drugs to combat COVID-19 using a signature-based approach

**DOI:** 10.1038/s41598-021-84044-9

**Published:** 2021-02-24

**Authors:** Sinead M. O’Donovan, Ali Imami, Hunter Eby, Nicholas D. Henkel, Justin Fortune Creeden, Sophie Asah, Xiaolu Zhang, Xiaojun Wu, Rawan Alnafisah, R. Travis Taylor, James Reigle, Alexander Thorman, Behrouz Shamsaei, Jarek Meller, Robert E. McCullumsmith

**Affiliations:** 1 Department of Neurosciences, University of Toledo College of Medicine and Life Sciences, Health Science Campus, Mail Stop #1007, 3000 Arlington Avenue, Toledo, OH 43614-2598 USA; 2 Department of Medical Microbiology and Immunology, University of Toledo, Toledo, OH USA; 3 Division of Biomedical Informatics, Cincinnati Children’s Hospital Medical Center, Cincinnati, OH USA; 4 Department of Biomedical Informatics, University of Cincinnati College of Medicine, Cincinnati, OH USA; 5 Department of Cancer Biology, University of Cincinnati College of Medicine, Cincinnati, OH USA; 6 Department of Environmental Health, University of Cincinnati College of Medicine, Cincinnati, OH USA; 7 Department of Electrical Engineering and Computing Systems, University of Cincinnati College of Medicine, Cincinnati, OH USA; 8 Department of Informatics, Nicolaus Copernicus University, Torun, Poland; 9 Neurosciences Institute, Promedica, Toledo, OH USA

**Keywords:** Virtual drug screening, Drug discovery

## Abstract

The COVID-19 pandemic caused by the novel SARS-CoV-2 is more contagious than other coronaviruses and has higher rates of mortality than influenza. Identification of effective therapeutics is a crucial tool to treat those infected with SARS-CoV-2 and limit the spread of this novel disease globally. We deployed a bioinformatics workflow to identify candidate drugs for the treatment of COVID-19. Using an “omics” repository, the Library of Integrated Network-Based Cellular Signatures (LINCS), we simultaneously probed transcriptomic signatures of putative COVID-19 drugs and publicly available SARS-CoV-2 infected cell lines to identify novel therapeutics. We identified a shortlist of 20 candidate drugs: 8 are already under trial for the treatment of COVID-19, the remaining 12 have antiviral properties and 6 have antiviral efficacy against coronaviruses specifically, in vitro. All candidate drugs are either FDA approved or are under investigation. Our candidate drug findings are discordant with (i.e., reverse) SARS-CoV-2 transcriptome signatures generated in vitro, and a subset are also identified in transcriptome signatures generated from COVID-19 patient samples, like the MEK inhibitor selumetinib. Overall, our findings provide additional support for drugs that are already being explored as therapeutic agents for the treatment of COVID-19 and identify promising novel targets that are worthy of further investigation.

## Introduction

Severe acute respiratory syndrome coronavirus 2 (SARS-CoV-2) is responsible for the first global pandemic in a decade, coronavirus disease 2019 (COVID-19)^1^. Initial reports of a novel SARS-like acute respiratory syndrome emerged in late 2019 from Wuhan, China^2^. Since then, COVID-19 has spread to over 150 countries and all continents except Antarctica^3,4^. At the time of writing, over 35 million people have been infected, over 7.5 million of these in the US, and more than one million deaths have been attributed to this outbreak globally^4^. Millions of additional infections are projected to occur globally in upcoming months^3,4^.

COVID-19 is less infectious than SARS-CoV-1 but more lethal than the common flu^1^ with an estimated mortality rate of 3.4%^2^. The incubation period, on average, is 5.2 days; in severe cases, the median time course from disease onset to death is 14 days^5^. While fever, cough, fatigue, and myalgias^6–10^ are common, mild presentations of COVID-19, the disease can fatally evolve into a severe pneumonia, complicated by acute respiratory distress syndrome, hypoxemic respiratory failure, and cytokine storm secondary to prolonged infection^8^. In addition to the significant medical burden imposed by this outbreak, it is estimated that the global economic cost of COVID-19 will be over $1 trillion in 2020^11^. The emotional toll on individuals will be incalculable, with prolonged quarantine policies restricting personal freedom and social contacts.

Current treatment is supportive and is focused on managing disease complications and secondary symptoms^12–14^. Drugs indicated for other infectious diseases, such as antiviral and antiparasitic therapies, have been used for COVID-19 patients, but there is a paucity of evidence supporting their efficacy^15^.

To address the need for new therapies for COVID-19, research has focused on drug repositioning, particularly the use of bioinformatic tools to identify novel drug candidates that can be safely and rapidly repurposed to treat this disease. Integrating the results of SARS-CoV-2 transcriptomic analyses generated in infected cell lines, human tissues and even organoids^16^ with computational approaches has proven especially fruitful. Such studies identified FDA-approved antivirals^17^ and a broad range of kinase inhibitors as potential candidate treatments for COVID-19^18–20^. Importantly, transcriptional analyses have provided additional support for the use of drugs like dexamethasone^17^ and chlorpromazine^19^ that are already undergoing clinical trial. Analyzing the transcriptional changes induced by SARS-CoV-2 infection has also offered significant insight into the genes and biological pathways^17,18,21–23^ that are altered in disease, implicating cellular inflammatory responses, particularly interferon pathways, in COVID-19^19,24,25^.

In the present study we apply a signature-based connectivity analysis^26–28^ utilizing the extensive chemical perturbagen “omics” datasets deposited in the Library of Integrated Network-based Signatures (LINCS) database^26,29,30^. LINCS is a repository for systematically generated gene signatures based on the L1000 assay^31^. These gene signatures reflect cellular perturbations in response to pharmacological treatments; LINCS contains datasets for over 22,000 small molecules (drugs) in various cell lines. Different small molecules that produce signatures composed of highly similar patterns of gene expression changes, or “concordant” signatures, reflect shared connections between small molecules.

Here, we apply a two-pronged approach to identify novel compounds for the treatment of COVID-19. First, we identify pharmacologic therapies that are effective in the treatment of pathogens in the coronavirus family, like SARS and Middle East Respiratory Syndrome (MERS), as well as other viral illnesses^32–36^. We then identify candidate drugs in the LINCS database that are highly concordant with current therapies. Simultaneously, we generate gene signatures from a SARS-CoV-2 infected human cell line transcriptomic dataset. We directly match disease signatures with discordant small molecule signatures, thereby identifying drugs that “reverse” the disease signature. Finally, we compile a list of drugs from these two approaches to identify high-yield candidate drugs that may have therapeutic utility in the treatment of COVID-19 and verify that the candidates can also be generated using COVID-19 patient sample (in vivo) transcriptome data^24,25^. Our findings include many approved drugs already under trial for COVID-19 as well as novel candidates that have yet to be explored clinically but show promising antiviral efficacy in vitro, suggesting that this approach is of utility in identifying candidate repurposable drugs for the treatment of COVID-19.

## Results

**Figure 1:**
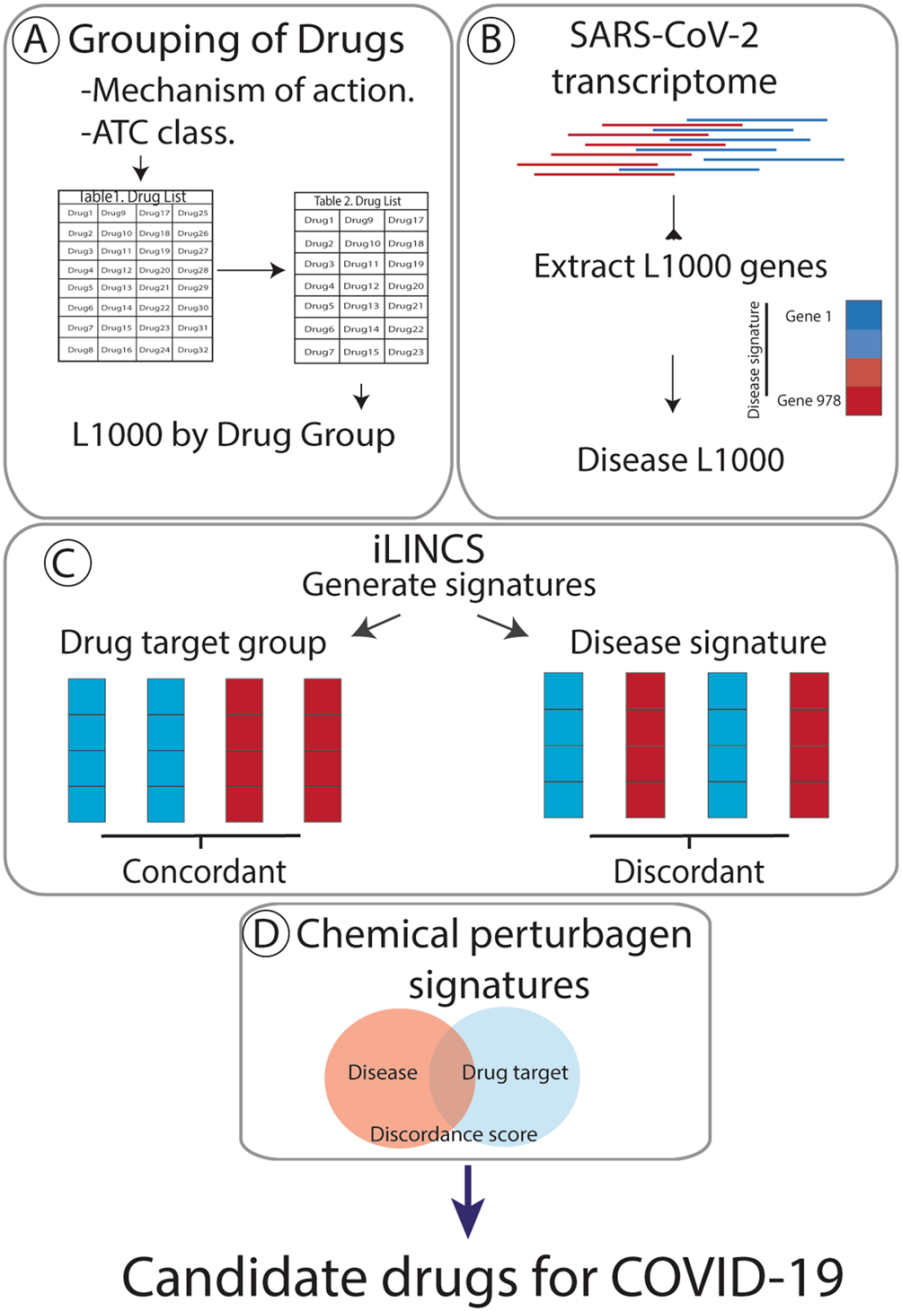
Overview of the workflow to identify candidate repurposable drugs to combat COVID-19. (**A**) Drugs that are currently in use to treat coronavirus and putative COVID-19 treatments were clustered based on mechanism of action and ATC class. (**B**) Gene expression data of the 978 genes that comprise the Library of Integrated Network-Based Cellular Signature (iLINCS) L1000 genes were extracted from severe acute respiratory syndrome coronavirus 2020 (SARS-CoV-2) (GSE147507) transcriptomic datasets. (**C**) Consensus iLINCS gene signatures were generated for drug groupings and disease. (**D**) Connectivity analysis was conducted and a list of chemical perturbagens that are concordant (≥ 0.321 concordance) to the drug target grouping signatures or discordant (≤ − 0.321 discordance) to the disease signatures was generated. Chemical perturbagens are filtered and curated to identify top candidate repurposable drugs.

Applying the workflow outlined in Fig. 1, we identified nine drugs, with known efficacy in treating coronavirus family pathogens, for which there are gene signatures in iLINCS. These drugs were clustered into five groupings according to their mechanism of action and Anatomical Therapeutic Chemical (ATC) classification (Table 1 and Table S1). Consensus gene signatures composed of genes changed LFC ≥ 0.85 and ≤ − 0.85 (Table S2) and combining data from 6 unique cell lines (Table S3) were generated for each drug cluster.

**Table 1:** Drug target groupings. Drug targets with iLINCS signatures that are in use or under investigation for the treatment of COVID-19 were grouped together if they met a least two of the three following criteria: Canonical Mechanism of Action, referenced from the database DrugBank (https://www.drugbank.ca/); Anatomic Therapeutic Chemical classification, referenced from https://www.whocc.no/atc_ddd_index/.

Drug Cluster	Drug	Canonical Mechanism of Action	Anatomical Therapeutic Chemical *First Level*
1	Chloroquine, Hydroxychloroquine	Toll-like receptor antagonists	Antiparasitic Products, Insecticides and Repellants
2	Lopinavir, Ritonavir	Protease inhibitors	Anti-Infective for Systemic Use
3	Fedratinib, Ruxolinitib, Bariticinib	JAK inhibitors	Antineoplastic and Immunomodulating Agents
4	Azithromycin	Macrolide antibiotic	Anti-Infective for Systemic Use
5	Losartan	Angiotensin receptor blocker antagonist	Cardiovascular System

**Figure 2:**
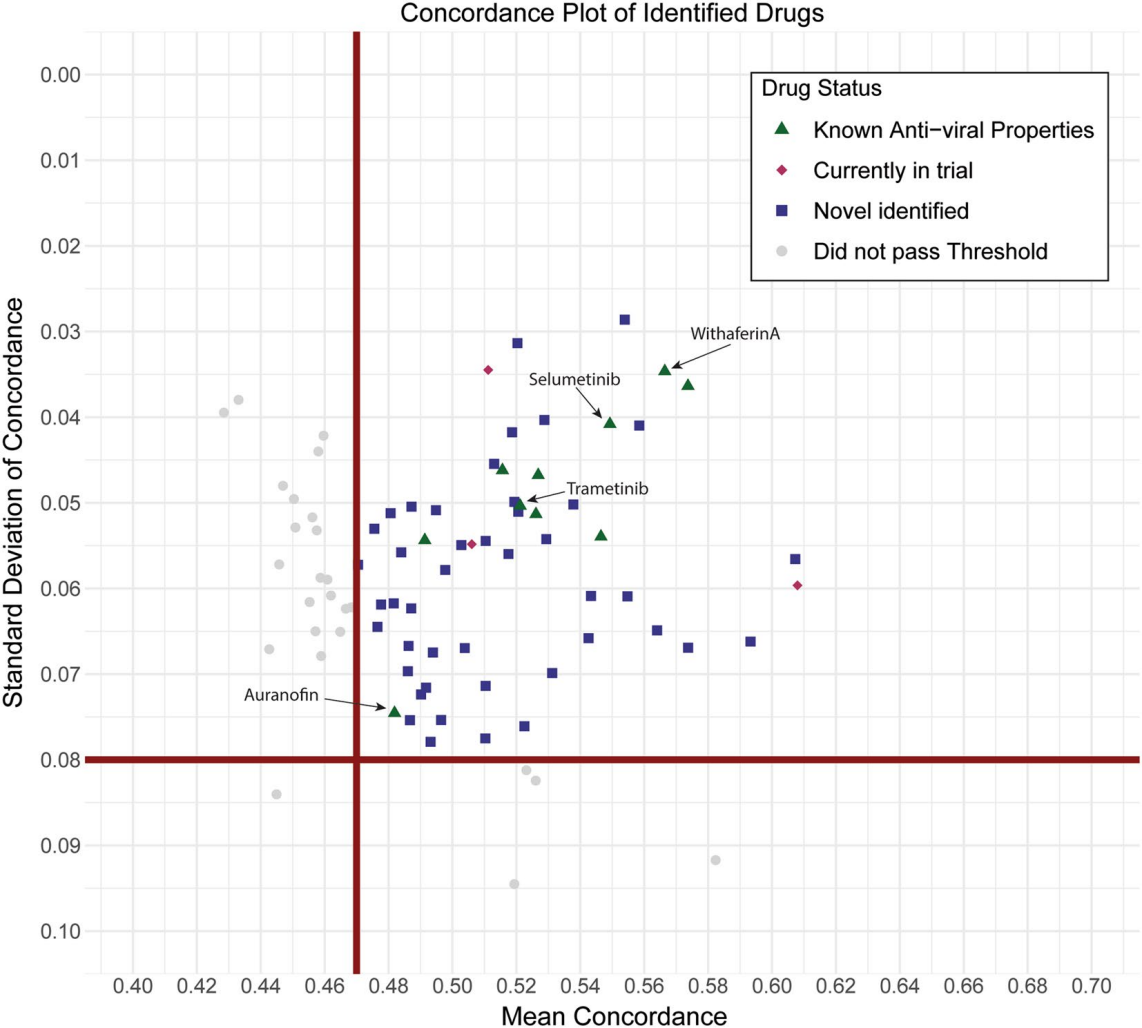
Scatter plot of average reported concordance scores and standard deviation (SD) of reported concordance scores for candidate drugs. A total of 83 FDA approved drugs were identified in the SARS-CoV-2 A549_ACE2 dataset (GSE147507 CL). 57 drugs were identified with a minimum mean concordance score 0.47and SD ≤ 0.08 (squares). Drugs above this threshold are considered “candidate” drugs. Top candidate drugs, those approved for use in humans and with demonstrated antiviral activity in vitro (filled triangles), those with SARS-CoV-2 antiviral efficacy specifically (inverted triangles) and those already in trial for COVID-19 (diamond) are also identified.

Simultaneously, we extracted differential gene expression data on the 978 genes that comprise the iLINCS L1000 from a publicly available SARS-CoV-2 infected cell line (A549_ACE2) transcriptomic dataset (GSE147507 CL). Consensus gene signatures composed of genes changed LFC ≥ 0.5 and ≤ -0.5 were generated for the SARS-CoV-2 signature (Table S4). In iLINCS, we conducted connectivity analysis to identify chemical perturbagens that are highly concordant to the drug target groupings (≥ 0.321) or highly discordant to the disease signature (≤ − 0.321), using established minimum iLINCS concordance score cutoffs^31,37^. This resulted in identification of 83 chemical perturbagens (Fig. 2). Fifty-seven chemical perturbagens were identified with a minimum mean concordance score 0.47 and SD 0.08 across all cell lines (Fig. 2).

Twenty chemical perturbagens were considered top “candidate” drugs for the treatment of COVID-19 (Table 2 and Table S5). The candidate drugs are FDA approved or are currently undergoing trial and are considered safe for human use and have reported antiviral properties in vitro. A subset of drugs has demonstrated antiviral properties against coronaviruses SARS-CoV, MERS or SARS-CoV-2. Seven of the 20 identified drugs are registered for clinical trial for the treatment of COVID-19 (clinicaltrial.gov).

**Table 2:** Top candidate drug findings for repurposing. Top candidate drug findings for repurposing. Candidate drugs are FDA-approved or currently undergoing trial; have reported antiviral properties and/or anticoronavirus properties (bold). Several of the candidate drugs identified for repurposing are already undergoing clinical trial for COVID-19. HCV hepatitis C virus; CHIKV Chikungunya virus; SFV Semliki Forest virus; RVFV Rift Valley fever virus. ** Also identified in 2 different COVID-19 patient datasets.

Drug	Drug class	Antiviral properties
**Trametinib**	Kinase inhibitor	MERS-CoV^38^, SARS-CoV-2^39^
**Withaferin A**	Steroidal lactone	SARS-CoV-2^40–43^
**Parthenolide**	Sesquiterpene lactone	SARS^44^
**Lapatinib**	Kinase inhibitor	SARS-CoV-2^45^
**Sorafenib**	Kinase inhibitor	SARS-CoV-2^46^
**Auranofin**	Gold salt	SARS-CoV-2^47^
Selumetinib**	Kinase inhibitor	SARS-CoV-2^39^
Erlotinib	Antineoplastic, tyrosine kinase inhibitor	HCV, RNA viruses, dengue, Ebola^48–50^
Alvocidib	CDK Inhibitor	HSV, HIV, Flu^51–56^
Quinacrine	Antimalarial	EMCV, poliovirus^57^
Vandetanib	Kinase inhibitor	Andes virus^57^
Dasatinib	SRC tyrosine kinase inhibitor	HIV^58,59^
Thioridazine	Phenothiazine	Ebola^60,61^, HCV^62^; CHIKV, SFV^63^; RVFV^64^
**Candidate repurposable drugs currently in trial for COVID-19**		
Gallocatechin Gallate	Antioxidant	
Decitabine	Antineoplastic, cytosine analogue	
Fenretinide	Antineoplastic and chemopreventive synthetic retinoid	
Curcumin	Anti-inflammatory, antimicrobial, antioxidant	
Simvastatin	Antilipemic	
Sirolimus	Macrolide lactams	
Cyclosporine	Immunosuppressant	

**Figure 3:**
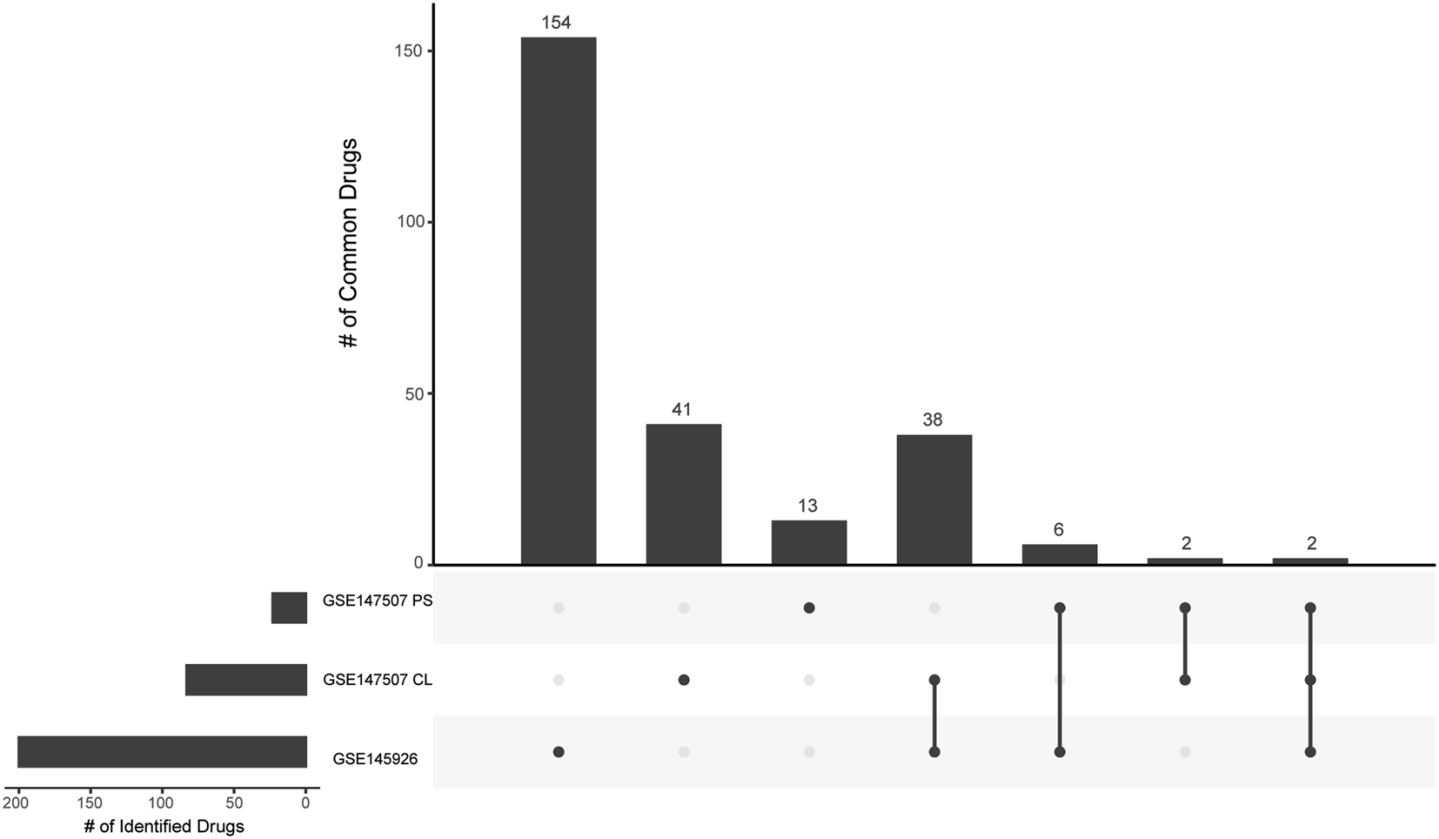
UpSet plot summarizing the overlap between the candidate drugs identified for three different COVID-19 disease signatures. Our primary dataset, GSE147507 CL: SARS-CoV-2 A549_ACE2 infected cell line samples described in Blanco-Melo et al^24^; GSE147507 PS: COVID-19 patient samples described in Blanco-Melo et al.^24^ and GSE145926: COVID-19 patient samples described in Liao et al^25^. The bar chart shows the number of unique and common candidate drugs across disease datasets, as indicated by matrix dots (# candidate drugs unique to a single dataset) or dots connected by lines (# candidate drugs common to at least 2 datasets). Figure generated using R package UpSetR^107^.

Although transcriptomic data is now available from COVID-19 infected patient tissues^24,25^, our approach generated the list of candidate drugs using gene signatures generated from SARS-CoV-2 infected A549_ACE2 cell lines from dataset GSE147507^24^. Chemical perturbagen gene signatures in iLINCS are also generated using cancer cell lines, including the A549 line. There was little correlation between iLINCS gene signatures generated from COVID-19 patient samples (GSE147507 PS and GSE145926) making it difficult to establish a baseline transcriptomic profile (Fig. S1). The clinical heterogeneity of the COVID-19 patient sample-derived transcriptomic signatures made the signature “noisier.” This may reflect the different tissue types analyzed in each study (postmortem lung tissue vs bronchoalveolar lavage fluid), the heterogeneity of SARS-CoV-2 infection/COVID-19 in individual patients, the small sample size and differences in RNAseq analysis methods. However, we confirmed that a subset of the candidate drugs identified from the SARS-CoV-2 infected cell line signature are also identified from the COVID-19 patient samples. Using the same gene signature approaches and cutoffs, we found that 4/23 candidate drugs we identified from the GSE147507 COVID-19 patient dataset and 40/200 candidate drugs we identified from the GSE145926 COVID-19 patient dataset were common with our list of 83 candidate drugs (Fig. 3).

Two drugs, selumetinib and radicicol, were common to both patient sample datasets and the SARS-CoV-2 (GSE147507 CL) dataset. Of particular interest is selumetinib, an FDA approved treatment for neurofibromatosis. As with many other drugs identified by this pipeline, selumetinib is a kinase inhibitor, acting as a non-ATP-competitive MEK1 and MEK2 inhibitor^65^. MEK inhibitors have known antiviral efficacy against coronaviruses, inhibiting the Raf/MEK/ERK signaling pathway and impairing viral production but not viral entry into the cell in a murine coronavirus hepatitis virus model^66^. It was recently shown that selumetinib, like trametinib, a MEK inhibitor also identified in this study, can stimulate natural killer cells, reduce ACE2 expression in human cells, and reduce cytokine expression in COVID-19 patient plasma, suggesting that this class of drugs may both suppress infection by SARS-CoV-2 and support the body’s immune response to infection^39^. Overall, our approach acts a useful screen for identifying common candidate drugs to reverse gene signatures for SARS-CoV-2 infected tissues despite the differences in disease transcriptome profiles from different data sources.

The drug clusters outlined in Table 1 are composed of drugs which showed initial promise for treating COVID-19 and/or are commonly found in computational or experimental studies searching for inhibitors of COVID-19^67^, but whose efficacy has not necessarily been confirmed clinically^68,69^. Due to concerns that utilizing these drug clusters may skew identification of candidate drugs, we also generated a candidate drug list using disease signatures only. We applied the workflow (using the same LFC thresholds) to generate candidate drugs from the SARS-CoV-2 cell line dataset only but did not apply the drug cluster filter. We confirmed that the candidate drugs identified from the SARS-CoV-2 infected cell line signature are a superset of the drugs identified after applying the drug cluster filter, suggesting the two pronged approach results in complementary results and does not skew the identified candidate drug list from SARS-CoV-2 relevant findings. We applied a relatively stringent drug cluster filter (candidate drugs must also be present in at least out of 2 of 5 drug cluster analyses) to our analysis. However this feature of the workflow can be modified by users as necessary, by reducing or increasing the stringency or entirely removing drug clusters as a filter. Removing this filter will increase the number of candidate drugs identified but may also result in an increased number of false positive discoveries. This filter can be modified in the workflow code deposited in GitHub. Other filters applied during analysis, such as mean concordance and standard deviation scores, can also be adjusted using the interactive document that accompanies this study (https://banseljaj.shinyapps.io/covid19_drugs_list/).

Thus, we distilled a list of drugs derived from pharmacological and disease perturbation signatures that may have therapeutic utility in the treatment of COVID-19. Top candidate drugs include trametinib, lapatinib, withaferin A, parthenolide, sorafenib and auranofin, which have demonstrated antiviral properties in vitro in the treatment of coronaviruses including SARS-CoV-2 but have not yet been explored clinically for the treatment of COVD-19. Candidate drugs gallocatechin gallate, decitabine, curcumin, fenretidine, cyclosporine, simvastatin and sirolimus are currently registered for clinical trial in the treatment of COVID-19. The remaining top candidate drugs identified by our analysis include kinase inhibitors erlotinib, alvocidib, dasatinib, antimalarial quinacrine, and the phenothiazine thioridazine which is more commonly used as an antipsychotic. These drugs also have antiviral properties and are yet to be explored for the treatment of COVID-19.

Finally, we conducted biological pathway analysis using Reactome, searching the same genes (LFC 0.5) that compose the gene signatures used in our workflow (Fig. S2). Three biological pathways were common to the SARS-CoV-2 infected cell line and both patient sample sets: Signaling by Interleukins, Interleukin-4 and Interleukin-13 signaling and Signaling by Receptor Tyrosine Kinases (Fig. S2D). As expected, immune related pathways like interleukin signaling have also been reported by others following analysis of SARS-CoV-2 infection datasets^18,19^. Our workflow (modified) has also provided in silico confirmation of the anti-inflammatory and pro-immune effects of oxytocin^70^ and the antidepressant fluoxetine^71^, which is also currently in trial for the treatment of COVID-19 (NCT04377308, and others). Interestingly, pathways related to Cell Cycle, and CDK and TP53 transcriptional regulation of cell cycle genes were predominately identified following analysis of the patient sample datasets and likely indicate changes in cell cycle regulation following SARS-CoV-2 infection^72^. In addition to immune dysregulation, biological pathway analysis also supports targeting viral replication processes in SARS-CoV-2 infection. Indeed, biological pathway analysis of genes that are significantly altered (LFC 1) by candidate drug selumetinib (identified as common to all disease datasets in this study), identified pathways that were also common to both patient sample datasets (Fig. S2F). These pathways are involved in cell cycle regulation: Transcriptional regulation by TP53, Mitotic Gi phase and G1/S transition, cell cycle, G1/S transition and Cell Cycle Mitotic (Fig. S2E). No common pathways to all 3 disease datasets (SARS-CoV-2 cell line and patient sample datasets) and selumetinib were found. Selumetinib is a MEK kinase inhibitor and as discussed above, can regulate the canonical Raf/MEK/ERK signal transduction pathway, potentially inhibiting viral replication. This pathway is utilized (hijacked) at different stages of the viral life cycles by many DNA and RNA viruses including coronavirus SARS-CoV-2^73^. This study provides additional support for the exploration of MEK inhibitors at treatments for COVID-19.

## Discussion

Vaccination programs for COVID-19 are progressing rapidly. However, the scale and cost of this global health crisis is such that effective drug therapies have an important and complementary role to play in treating this disease. In recent months, in silico studies have identified putative repurposable drugs for treating COVID-19^20,50,74–77^. Many of these studies exploit the finding that SARS-CoV-2 may enter the cell by binding to angiotensin converting enzyme 2 (ACE2)^78^ and utilize a combination of structural and biomedical data to identify drug candidates^20^. To advance therapeutic discovery and identify the most promising candidate drugs for COVID-19, we employ an alternative, signature-based bioinformatic approach.

In this study, we data mine the extensive LINCS database, which acts as a repository of “L1000” gene signatures generated by treating various cell lines with over 20,000 small molecules. The L1000 genes are a reduced representation of the transcriptome, a method by which a select group of genes account for ~ 82% of the information content of the transcriptome^79^. The approach involved feature selection/reduction techniques applied to 12,063 gene expression samples profiled on microarrays from GEO^80^. Benchmarking of the L1000 assay versus RNAseq yielded a cross-platform correlation of 0.84^79^, suggesting the L1000 assay represents an efficient alternative to RNAseq.

Utilizing this resource, our two-pronged connectivity analysis approach identified candidate drugs that are (1) highly concordant to current drugs employed to treat coronavirus family pathogens and (2) highly discordant to SARS-CoV-2 transcriptomic signature. Seven of the identified candidate drugs are already registered for clinical trial (clinicaltrials.gov) as therapies for COVID-19. This includes the immunosuppressants sirolimus. Sirolimus was identified in our study and another in silico drug screen^74^ as a candidate repurposable drug for treating COVID-19. Immunosuppressants may address the symptoms resulting from overactivation of the immune system (“cytokine storm”) in response to COVID-19 infection^81^. Our screen also identified the immunosuppressant thalidomide, although at a less stringent cutoff (SD 0.081; outside cutoff threshold). Thalidomide is a potent anti-inflammatory, approved by the FDA for treatment for multiple myeloma and erythema nodosum leprosum, an immune-mediated complication of leprosy^82^. Although concerns regarding the wide-spread adoption of thalidomide as a treatment of COVID-19 have been raised, due in part to the potential side-effects^82^, clinical trials to assess efficacy and safety as a treatment for COVID-19 commenced following publication of a case report of the protective effect of thalidomide on immune dysfunction and lung injury in a single patient^83^. These findings support the utility of using this transcriptomic signature based approach to identify repurposable drugs for treating COVID-19, and lend further support to explore these promising candidate drugs. Indeed, seven of the top candidate drugs we identified have shown antiviral efficacy for coronaviruses or SARS-CoV-2, specifically, in vitro.

Lapatinib blocked SARS-CoV-2 cytopathic effect and viral infection as assessed by viral RNA accumulation, and prevented accumulation of N protein in MRC5 (human pulmonary fibroblast cell line) cells expressing ACE2 that were infected with SARS-CoV-2^45^. The concentration of lapatinib required to inhibit SARS-COV-2 in this study can be achieved in human tissue at currently prescribed doses^84^. Lapatinib is a dual inhibitor of epidermal growth factor receptor and human epidermal growth factor receptor (HER2) tyrosine kinases^85^. However, its antiviral efficacy was thought to result from an alternative mechanism, via inhibition of the SARS-CoV-2 protease 3CLpro, as determined by molecular docking approaches^86^. Different experimental reports suggest that lapatinib may inhibit 3CLpro activity^87^ or has no effect on this protein^86^. The MEK inhibitor trametibinib displayed strong inhibitory activity against MERS-CoV infection in Huh7 human hepatocytes when administered prior to and post-infection, suggesting that ERK/MAPK pathway signaling may be important for viral entry and viral replication stages of the MERS-CoV life cycle^38^. As with the candidate drug selumetinib which was one of the only drugs common to both of the patient COVID-19 sample analyses and the SARS-CoV-2 analysis, trametinib also shows efficacy against SARS-CoV-2 in vitro^39^, highlighting growing interest in the antiviral potential of this class of drug. Withaferin A has anti-inflammatory and anti-tumor properties^43,88,89^ and may be useful in targeting the pathological immune component associated with COVID-19 infection. Molecular docking approaches predicted that withaferin A binds and blocks cell surface receptors like transmembrane protease serine 2 (TMPRSS-2), which are required for virus entry into host cells^41,42^. Withaferin A may act in a similar manner as the serine protease inhibitor camostat mesylate^41^, binding and blocking the catalytic site of transmembrane protease serine 2 (TMPRSS-2) which is required for priming of the SARS-CoV-2 S protein, thus preventing SARS-CoV-2 infection of lung cells^90^. Auranofin is an FDA-approved gold-containing triethyl phosphine used to treat rheumatoid arthritis^91^. Auranofin treatment of SARS-CoV-2 infected cells resulted in a significant reduction in viral RNA at 24hrs and 48hrs and SARS-CoV-2 infectivity titers at 48hrs post infection^47^. Although its mechanism of action in SARS-CoV-2 infection is not known, auranofin is an inhibitor of redox enzymes which leads to oxidative stress and cell apoptosis^92^ and also acts as an anti-inflammatory by inhibiting JAK1 and STAT3 phosphorylation and IL-6 signaling^93^. Interestingly, cytokine (IL-6, IL1β, TNFα) expression was also significantly reduced in auranofin treated SARS-CoV-2 infected cells following auranofin treatment at both 24 h and 48 h time points. Sorafenib is a multikinase inhibitor that was also identified in a large scale in vitro drug screen of candidate repurposable drugs for COVID-19^46^. Although identified as an active compound against SARS-CoV-2, the low selectivity index (SI = 1) poses significant concerns about whether a sufficient concentration can be safely administered^46^. Pharmacokinetic and safety data is available for this FDA-approved renal cell carcinoma treatment but this study highlights the importance of screening and assessing novel candidate drug treatments, particularly antineoplastics, for safety as well as efficacy. In a study reported in bioRxix, the CDK kinase inhibitor alvocidib, an investigational antineoplastic explored as a treatment for small-cell lung cancer, prevented cytopathic effects in SARS-CoV-2 infected VeroE6 cells, but also had unfavorable cytotoxicity at the effective concentration^94^, suggesting that the potential toxicity of some antineoplastic drugs may diminish their utility as therapies for COVID-19.

Additional candidate drugs identified have demonstrated antiviral, but not necessarily anti-coronavirus properties. The main class of drugs identified from our analyses are kinase inhibitors. Kinase inhibitors are high-yield targets, with new small molecule kinase inhibitors being developed every year and over two dozen small molecule kinase inhibitors already approved for human use^95^. Their potential as antiviral treatments has also been explored in recent years^50,96–98^. Viruses depend on host cell protein kinases for every step of their life cycle, including viral entry into the cell, cell cycle processes and cellular stress response^99^. Thus, targeting these protein kinases using kinase inhibitors will disrupt the virus’s ability to hijack cellular processes. As many host protein kinases are broadly required by different viruses, kinase inhibitors are excellent candidates for broad-spectrum antiviral therapies^97^. Kinase inhibitors represent an expanding, if underexplored, avenue of research for the treatment of viral illnesses, including coronaviruses. Repurposing kinase inhibitors, many of which are already approved for use in humans as cancer treatments, is a time-and cost-effective method to identify new therapeutics in a rapidly evolving situation such as the one posed by the current outbreak of COVID-19.

## Limitations

The antimicrobial drugs that comprise our drug target groupings are limited to those that have gene signatures in iLINCS. As with other in silico screening approaches, the candidate drugs identified here are not necessarily ready for human use. These candidate drugs were initially identified from LINCS gene signatures generated in cancer cell lines which may not reflect the microenvironment of human tissues infected with SARS-CoV-2. However, we later confirmed a subset of the identified drugs were also found following analysis of two different transcriptome datasets generated from COVID-19 patient samples. Several of the candidate drugs are used in the treatment of viral infections but not SARS-COV-2 or COVID-19 specifically, and require further investigation for dosage, efficacy etc. before they can be used in humans.

In summary, our approach has identified candidate repurposable drugs, from the > 20,000 small molecules in the LINCS repository, that may be utilized to combat COVID-19. Several of the identified drugs are already registered for clinical trial for the treatment of this illness. The candidate drugs are also (1) safe for use in humans, ((2) have demonstrated antiviral efficacy in vitro*,* including against coronavirus pathogens and (3) are discordant for SARS-CoV-2 disease signature. Thus, our results provide additional support for candidate drugs that are currently undergoing trial or are of interest to researchers. Our findings also contribute to the relatively novel literature addressing the purported broad-spectrum antiviral efficacy of kinase inhibitors and may offer a novel avenue for investigation in the search for COVID-19 therapies. While there is evolving evidence for kinase inhibitors as antivirals, other antimicrobials could be repurposed as well.

## Methods

### Selecting and grouping antimicrobials with known efficacy in treating coronavirus family pathogens

The workflow for this study is outlined in Fig. 1. Analysis was conducted using R^100^. We conducted a PubMed search using search terms “coronavirus” or “COVID-19” and “antiviral” or “drug” or “therapy” and generated a list of compounds utilized to treat coronavirus family pathogens or identified as putative COVID-19 therapeutics. We identified seventeen drugs for potential analysis (Table S1). L1000 gene signature datasets were available for nine of the seventeen drugs (Table 1) using the integrative web platform iLINCS (http://ilincs.org). The iLINCS L1000 hub gene assay assesses genome-wide transcriptional changes following perturbation by one of more than 20,000 small molecules^79^. Eight drugs without signatures were excluded from further analysis. Gene signatures were generated for all 9 remaining drugs. To standardize our analysis, we combined gene signature data from 6 different cell lines for each drug. Where possible, signatures for a 24-h time point and 10 µM concentration conditions were used. The cell lines and conditions are listed in Table S3. Data from cell lines were used if gene signatures for at least 6 of the 9 drugs were available for that cell line.

Next, we grouped the nine drug targets based on canonical mechanism of action and the Anatomical Therapeutic Chemical (ATC) classification. The database DrugBank (https://www.drugbank.ca/) was used to group the drugs by their canonical mechanisms of actions. Drug identification was only referenced from Drug Bank I.D. If no Drug Bank I.D. was available, this is indicated in Table 1 and Table S1. If there was no listed MOA from Drug Bank, then the MOA was appropriately cited, referenced from iLINCS, or was referenced from Gene Ontology (GO) Molecular Function 2018 accessed via Enrichr (http://amp.pharm.mssm.edu/Enrichr/enrich). Next, drugs were classified based on the ATC classification system (https://www.whocc.no/atc_ddd_index/). If a particular drug did not have an ATC classification, it was marked as “unclassified.” From DrugBank, we also collected the clinical indications, gene targets, and trade names. In addition, we probed the ATC Index (https://www.whocc.no/atc_ddd_index/) to identify the first- and second-level of drug classifications. The first-level classification was used to confirm drug grouping. With a final list of drug clusters, the individual drug signatures within each grouping were collected and averaged across the L1000.

### Generating iLINCS gene signatures

To generate all consensus gene signatures (drug cluster and disease signatures), L1000 genes with a minimum log fold change (LFC) in expression were selected. The use of LFC is an established and reproducible method for selecting biologically relevant gene changes in transcriptomic datasets^101–104^. The optimal LFC threshold for each dataset was determined after examining the number of chemical perturbagens identified at 5 different thresholds: all L1000 genes, LFC 0.26, LFC 0.5, LFC 0.85 and LFC1*.* Optimal LFC thresholds were selected to reduce excess noise (non-specific gene data) from the analysis without applying overly stringent cutoffs, factors that may curtail identification of candidate drugs. Different thresholds were applied to generate consensus gene signatures for drug cluster and disease signatures. Experimentally, drug cluster signatures are generated by applying chemical perturbagens to cancer cell lines and assaying the L1000 (978 genes). Disease signatures are generated by extracting the L1000 gene data from RNAseq analysis of SARS-CoV-2 infected cells or tissues. Thus, the same LFC thresholds may not be optimal for all datasets, particularly those generated under such different conditions.

### Generating iLINCS gene signatures for drug clusters

Using the iLINCS portal, we acquired the LINCS chemical perturbagen signatures (978 genes that comprise the L1000) for each drug candidate. Genes with a LFC value of ≥ 0.85 or ≤ − 0.85, indicating differential gene expression induced by the drug target compared to a corresponding control cell line, were identified. This threshold was selected after examining the number of chemical perturbagens identified at 5 different thresholds: all L1000 genes, LFC 0.26, LFC 0.5, LFC 0.85 and LFC 1.0 (see Table S2). We also tested the symmetric distribution of genes identified at ≤ -0.85 and ≥ 0.85 LFC for each drug cluster in each cell line to confirm that similar numbers of downregulated and upregulated genes were included in consensus gene signatures*.* Our L1000 consensus gene signatures follow an approximately symmetric normal distribution in every case*.* Thus, a uniform cutoff of LFC ≤ − 0.85 and ≥ 0.85 gives us approximately the same number of genes across the distribution.

Gene lists were pooled and averaged such that a master list of differentially expressed genes was generated for each drug candidate family. For example, genes with a LFC ≥ 0.85 or ≤ − 0.85 that appeared in both the hydroxychloroquine gene signature and the chloroquine gene signature were averaged to calculate mean values for each differentially expressed gene in drug target grouping 1. The upregulated genes (LFC ≥ 0.85) were clustered and the downregulated genes (LFC ≤ − 0.85) were clustered. These clusters were uploaded as user generated signatures into iLINCS. Next, we identified connected chemical perturbagens, utilizing a concordance threshold score of ≥ 0.321, an established minimum concordance score cutoff^31,37^, to identify chemical perturbagen signatures that are considered highly correlated with our drug target grouping signatures.

### Generating iLINCS gene signatures coronavirus-family induced disease datasets

We utilized SARS-COV-2 transcriptomic data from three different datasets in this study. Our primary analysis were conducted using SARS-CoV-2 (GSE147507 CL) RNAseq data generated in A549_ACE2 expressing cells (n = 3), an adenocarcinomic human alveolar basal epithelial cell line that overexpress receptor ACE2 required for viral entry into the cell and mock-treated A549_ACE2 expressing cells (n = 3), one of several SARS-CoV-2 transcriptomic datasets generated by Blanco-Melo et al. in their study^24^. For confirmation analysis we used SARS-CoV-2 RNAseq data from COVID-19 patient postmortem lung samples (n = 2) and healthy lung biopsies (n = 2) generated in the same study (GSE147507 PS)^24^, and a dataset generated from single cell RNAseq analysis of bronchoalveolar lavage fluid immune cells from moderate, severe and critically ill patients with COVID-19 (n = 9) and healthy controls (n = 3) (GSE145926)^25^.

We conducted differential gene expression analysis of the GSE147507 cell-line dataset comparing SARS-CoV-2 infected samples to corresponding mock-treated control samples. RNASeq raw count data was analyzed in R Software (v 4.0.1) (R Software Foundation) using the edgeR R Package (v3.30.3). For quality control, we used the built-in function filterByExpr that only keeps the genes with a high enough count across all samples as calculated by the strategy of Chen et al.^105^. Normalization was performed using the calcNormFactors() method with the Trimmed mean of M-Values method.

Following analysis of the SARS-CoV-2 transcriptomic dataset, the subset of genes that comprise the LINCS L1000 were extracted. The extracted L1000 genes were uploaded into iLINCS. Genes with LFC in expression within four thresholds, 0.26 LFC, 0.5 LFC, 0.85 LFC, LFC 1 and all L1000 genes, were identified with a custom R script for further processing. The optimal LFC cutoff was determined as a LFC ≥ 0.5 or ≤ − 0.5 following examination of the number of chemical perturbagens identified with consensus gene signature at this threshold (Table S4).

As described above, upregulated and downregulated disease gene signatures were generated for each disease dataset (within each threshold) and uploaded into iLINCS to identify connected perturbagens. For disease gene signatures, chemical perturbagen signatures that are highly discordant (discordance score ≤ − 0.321), indicating these perturbagens may “reverse” the disease signature, were identified. Genes at LFC ≥ 0.5 and ≤ − 0.5 threshold were then carried forward for further analysis. Utilizing this gene threshold generated optimal SARS-COV-2 disease signatures to identify a large number of discordant chemical perturbagens.

### Identification of candidate chemical perturbagens (drugs) to treat COVID-19

Candidate drugs were identified from the chemical perturbagen connectivity analysis using a custom script in R^100^ and figures were produced using the package ggplot2^106^ (Fig. 2) and package UpSetR^107^ (Fig. 3). The script downloaded the data from the iLINCS API and used the following criteria: Chemical perturbagens had a concordance score ≥ 0.321 compared to drug target grouping signatures or a discordance score ≤ -0.321 compared to disease signature. If the same chemical perturbagen is identified multiple times, from different experimental conditions, replicate findings are removed so that only the highest concordance score (or lowest discordance score) for each chemical perturbagen remains. 168 chemical perturbagens were identified in the SARS-CoV-2 (GSE147507 CL) disease signature analysis AND at least 2/5 drug target grouping signature analyses. Following a crude filter step to identify FDA approved drugs, this resulted in 83 candidate chemical perturbagens identified. We took the mean and standard deviation of the concordance values of each candidate chemical perturbagen across all cell line combinations. The resulting data presented in Fig. 2 and accessible for exploration in an interactive document (https://banseljaj.shinyapps.io/covid19_drugs_list/), allowed us to identify the drugs with the highest concordances (high mean) and minimum level of disagreement between cell lines (low SD). We chose the cutoff of ≥ 0.47 for mean and ≤ 0.08 for standard deviation, resulting in a shortlist of 57 candidate drugs. A final list of 20 candidate drugs which are FDA approved (or under trial) and have antiviral properties are considered top hits.

We confirmed that the candidate drugs identified using our workflow were also discordant with SARS-CoV-2 disease signatures generated from COVID-19 patient samples. We accessed transcriptomic data generated from COVID-19 patient samples (GSE147507 PS; GSE145926), extracted the L1000 and generated a consensus gene signature using the same approach described above (threshold LFC ≥ 0.5 or ≤ -0.5). We identified 23 FDA-approved candidate drugs that were discordant with the Mt. Sinai GSE147507 PS COVID-19 patient disease signature and 200 candidate drugs that were discordant with the GSE145926 disease signature. We looked at the intersection of the gene signatures for all 83 identified candidate drugs from our initial analysis (GSE147507 CL) and the COVID-19 GSE147507 PS and GSE145926 disease signatures. We found that 4/23 and 40/200 of these drugs were common to our primary analysis (Fig. 3). Two drugs were common to both patient datasets only.

### Biological pathway analysis

Using the Reactome pathway database^108^, we searched the genes with significantly altered expression (LFC > 0.5), the same gene sets used to generate the SARS-CoV-2 cell line and patient sample disease signatures (GSE147507 CL, GSE147507 PS and GSE145926), to identify the top 15 biological pathways altered following SARS-CoV-2 infection. We also identified the top 15 biological pathways for candidate drug selumetinib, using genes with significantly altered expression (LFC + /− 1) obtained from an A549 treated cell line (24 h, 10uM concentration) from the iLINCS database. Venn diagrams showing the intersection of the biological pathways altered by disease and drugs were drawn using the webtool available at bioinformatics.psb.ugent.be/webtools/Venn/.

### GitHub repository access

The complete workflow of this analysis has been deposited in github and can be accessed at https://github.com/AliSajid/Covid19/tree/v1.10. This analysis was conducted using v1.10 of the repository that has been deposited (https://doi.org/10.5281/zenodo.4439441).

## Supplementary Information

Supplementary Information

## Data Availability

The datasets analyzed during the current study are available in the Gene Expression Omnibus (GEO: https://www.ncbi.nlm.nih.gov/geo/) (GSE56192; GSE47963; GSE147507) and the Library of Integrated Network-Based Cellular Signatures (LINCS) via iLINCS (http://ilincs.org).

## Code availability

R scripts can be accessed via: https://github.com/AliSajid/Covid19/tree/v1.10.
