# Supplementary material for: Identification of candidate repurposable drugs to combat COVID-19 using a signature-based approach
Source: Sci Rep. 2021 Feb 24;11:4495. doi: 10.1038/s41598-021-84044-9 (PMC7904823; doi:10.1038/s41598-021-84044-9)
Supplement: Supplementary file 1 — Supplementary Information [file 41598_2021_84044_MOESM1_ESM.pdf]

**Supplementary Information.**

# Identification of candidate repurposable drugs to combat COVID-19 using a signature-based approach

Sinead M O'Donovan, Ali Imami, Hunter Eby, Nicholas D Henkel, Justin Fortune Creeden, Sophie Asah, Xiaolu Zhang, Xiaojun Wu, Rawan Alnafisah, R. Travis Taylor, James Reigle, Alexander Thorman, Behrouz Shamsaei, Jarek Meller, Robert E McCullumsmith

| Drug | Trade Names | Clinical Indication | Target | Canonical Mechanism of Action and Clusters | Anatomical Chemical Therapeutic Classification | Literature Citing Relevance to SARS-CoV-2 and Coronaviridae | Putative Effect on SARS-CoV-2 |
|---|---|---|---|---|---|---|---|
| ***Drugs with iLINCS signature present.*** | | | | | | | |
| Hydroxy-chloroquine [1] | Plaquenil | Malaria; Immuno-suppressant | Toll-like receptor 9; Toll-like receptor 7 | Toll-like receptor antagonist | Antiparasitic products, insecticides and repellents | [2-4] | Alkalization of phagolysosome; modification of post-translation protein modifications [5,6] |
| Chloroquine [7] | Aralen | Malaria; Amebiasis | Toll-like receptor 9 Glutathione S-transferase (A2); High mobility group protein B1; tumor necrosis factor | | | | |
| Lopinavir [8] | Kaletra / Aluvia (with Ritonavir) | HIV/AIDS Antiviral Therapy | Human immunodeficiency virus type 1 protease | Protease inhibitor | Antiinfectives for systemic use | [9-12] | Inhibition of CoV-polyprotein processing [9,13] |
| Ritonavir [14] | Norvir; Kaltera /Aluvia (with Lopinavir) | | | | | | |
| Fedratinib [15] | Inrebic | Antineoplastic | Tyrosine-protein kinase JAK2; Receptor-type tyrosine-protein kinase FLT3 | JAK Inhibitor | Antineoplastic and immunomodulating agents | [16] | Inhibition of clathrin-mediated viral endocytosis; mediation of inflammatory response, attenuation of cytokine storms from prolonged infection [16,17] |
| Ruxolitinib [18] | Jakafi, Jakavi | Antineoplastic | Tyrosine-protein kinase JAK1 and JAK2 | | | | |
| Baricitinib [19] | Olumiant, Baricinix | Immuno-suppressant | Tyrosine-protein kinase JAK1, JAK2, and JAK3; Protein-tyrosine kinase 2-beta | | | | |
| ***Drugs with iLINCS signature present but not included in analysis.*** | | | | | | | |
| Azithromycin [20] | Act Azithromycin, AzaSITE, Zithromax, Zmax | Bacterial infections | 23S Ribosomal RNA; Protein-arginie deaminase type-4 | Inhibition of bacterial protein synthesis | Antiinfectives for systemic use | [4] | Adjunct therapy with hydroxychloroquine. |
| Losartan [21] | Act Losartan, Cozaar | Hypertension | Type-1 angiotensin II (AGII) receptor | AGII receptor antagonist | Cardiovascular system | [22] | Angiotensin receptor blocker. |
| ***Drugs with iLINCS signature not present.*** | | | | | | | |
| Remdesivir [23] | Remdesivir | Ebola | Replicase polyprotein 1ab; RNA-directed RNA polymerase L | Nucleoside analog, inhibition of viral RNA polymerase | No record | [24-27] | Inhibition of RNA dependent RNA polymerase [27] |
| Rocaglate | Not found in DrugBank. | | | | No record | [28] | Inhibition of viral translation [28]. |
| Silvesterol | Not found in DrugBank. | | | | No record | [28,29] | Inhibition of viral translation [28]. |
| Umifenovir [30] | Arbidol | Viral prophylaxis; Broad spectrum antiviral | Cytochrome P450 3A4, 2E1, 1A2, 2D6, 2C9, 3A5; UDP-glucuronosyltransferase 2B7, 1-9; Dimethylaniline monooxygenase 1, 3 | Aromatic rings interfere with viral endocytosis, exocytosis, intracellular trafficking, and destabilize membranes | Antiinfectives for systemic use | [31-33] | No specific purported mechanism yet published. |
| N10169 | Not found in DrugBank. | | | | No record | [34] | Inhibition of pyrimidine biosynthesis [34]. |

| | | | | | | | |
|---|---|---|---|---|---|---|---|
| Interferon Alphacon-1 [35] | Infergen | Antineoplastic | Interferon alpha/beta receptor 1, 2 | Interferon receptor agonist, JAK activator | Antineoplastic and immunomodulating agents | [36,37] | Upregulation of MHC 1 to present viral antigens. |
| *Polygonaceae spp.; Rheum palmatum L.* | Not found in DrugBank. | | | | No record | [38,39] | Inhibition of viral cell-entry by preventing interaction of CoV Spike Protein from interacting with ACE2 receptor [39] |
| Camostat mesylate [40] | N/A | Pancreatitis, Japan [41,42] | N/A | Serine protease inhibitor TMPRSS2 | Blood and blood forming organs | [43-45] | Inhibition of viral cell-entry [43] |

**Table S1.** Selected drugs implicated in the treatment of COVID-19.

Information relating to these drugs (trade name, clinical indication, targets, and canonical mechanism of action) were referenced from DrugBank (https://www.drugbank.ca/). Anatomical Therapeutic Chemical (ATC) classification was referenced from (https://www.whocc.no/atc_ddd_index/). The first-level of ATC classification is indicated.

**Table S2.**

| | | No. chemical perturbagens identified | | | | |
|---|---|---|---|---|---|---|
| | | Log fold change | | | | |
| **Cell line** | **Drug grouping** | **0** | **0.26** | **0.5** | **0.85** | **1** |
| A549-10uM-24h | 1 | 174 | 382 | 934 | 3528 | 5587 |
| A549-10uM-24h | 2 | 810 | 268 | 751 | 2449 | 3780 |
| A549-10uM-24h | 3 | 2002 | 2200 | 2474 | 3516 | 3799 |
| A549-10uM-24h | 4 | 701 | 961 | 1648 | 3743 | 4786 |
| A549-10uM-24h | 5 | 434 | 527 | 1455 | 4512 | 4612 |
| A549-10uM-6h | 1 | 997 | 1651 | 3134 | 4824 | 5090 |
| A549-10uM-6h | 2 | 505 | 655 | 1170 | 3455 | 4864 |
| A549-10uM-6h | 3 | 490 | 876 | 1313 | 2110 | 3009 |
| A549-10uM-6h | 4 | 378 | 558 | 859 | 2026 | 2465 |
| A549-10uM-6h | 5 | 1078 | 327 | 429 | 1422 | 3074 |
| HA1E-10uM-24h | 1 | 539 | 1860 | 4299 | 5477 | 5749 |
| HA1E-10uM-24h | 2 | 344 | 784 | 1724 | 3791 | 4078 |
| HA1E-10uM-24h | 3 | 662 | 953 | 1434 | 2804 | 3390 |
| HA1E-10uM-24h | 4 | 1054 | 1159 | 1468 | 1731 | 1890 |
| HA1E-10uM-24h | 5 | 314 | 600 | 1370 | 3713 | 4989 |
| HT29-10uM-24h | 1 | 539 | 1701 | 4286 | 5422 | 5557 |
| HT29-10uM-24h | 2 | 817 | 1113 | 2619 | 4173 | 4449 |
| HT29-10uM-24h | 3 | 2107 | 1660 | 1442 | 1963 | 2267 |
| HT29-10uM-24h | 5 | 713 | 1945 | 4633 | 6124 | 6170 |
| MCF7-10uM-24h | 1 | 452 | 1030 | 2923 | 5674 | 5783 |
| MCF7-10uM-24h | 2 | 378 | 729 | 1376 | 4493 | 5636 |
| MCF7-10uM-24h | 3 | 1890 | 2122 | 2119 | 3110 | 3420 |
| PC3-10uM-24h | 1 | 551 | 2394 | 6470 | 6567 | 6050 |
| PC3-10uM-24h | 2 | 1218 | 2114 | 3751 | 6381 | 6012 |
| PC3-10uM-24h | 3 | 1743 | 1875 | 1966 | 3172 | 4234 |
| PC3-10uM-24h | 4 | 880 | 380 | 822 | 3590 | 4879 |
| VCAP-10uM-24h | 1 | 2009 | 1175 | 1739 | 4745 | 5077 |
| VCAP-10uM-24h | 2 | 957 | 1742 | 3773 | 6912 | 6475 |
| VCAP-10uM-24h | 3 | 1630 | 1170 | 1865 | 3563 | 4568 |
| VCAP-10uM-24h | 4 | 1374 | 2314 | 3099 | 4148 | 4383 |
| VCAP-10uM-24h | 5 | 218 | 582 | 2073 | 5518 | 5532 |
| VCAP-10uM-6h | 1 | 2188 | 858 | 1210 | 4543 | 6498 |
| VCAP-10uM-6h | 2 | 1200 | 2315 | 3905 | 4855 | 4879 |
| VCAP-10uM-6h | 3 | 400 | 474 | 551 | 1562 | 2207 |
| VCAP-10uM-6h | 4 | 1065 | 841 | 955 | 1044 | 1612 |
| VCAP-10uM-6h | 5 | 3508 | 1151 | 1151 | 2552 | 4509 |

**Table S2.** L1000 genes with expression change at log fold change (LFC) threshold 0.85 were selected to generate drug cluster gene signatures. At this threshold, following connectivity analysis, a large number of candidate drug signatures were identified as concordant to the drug cluster signatures which represent drugs that are used or are being explored for the treatment of coronavirus or COVID-19. Higher thresholds (LFC 1) were found to be too permissive for chemical perturbagen identification, introducing excess noise, while lower thresholds (LFC 0.5-all LINCS signature) were more restrictive, limiting the number of chemical perturbagens identified.

**Table S3.**

| Cell line | Concentration | Time | No. of drug signatures | No. of unique drug signatures |
|---|---|---|---|---|
| A549 | 10uM | 24h | 9 | 6 |
| A549 | 10uM | 6h | 7 | 6 |
| HA1E | 10uM | 24h | 9 | 6 |
| HT29 | 10uM | 24h | 9 | 7 |
| MCF7 | 10uM | 24h | 15 | 6 |
| PC3 | 10uM | 24h | 10 | 6 |
| VCAP | 10uM | 24h | 9 | 7 |
| VCAP | 10uM | 6h | 8 | 7 |

**Table S3.** iLINCS cell lines and conditions used to generate signatures for 9 drugs used for drug clustering analysis.

**Table S4.**

| | | No. chemical perturbagens identified | | | | |
|---|---|---|---|---|---|---|
| | | log fold change (LFC) | | | | |
| **Cell line** | **Disease** | **0** | **0.26** | **0.5** | **0.85** | **1** |
| A549-10uM-24h | GSE145926 | 310 | 457 | 846 | 3544 | 3472 |
| A549-10uM-24h | GSE147507 PS | 16 | 32 | 83 | 293 | 394 |
| A549-10uM-24h | GSE147507 CL | 68 | 168 | 387 | 2371 | 4596 |
| A549-10uM-6h | GSE145926 | 310 | 457 | 846 | 3544 | 3472 |
| A549-10uM-6h | GSE147507 PS | 16 | 32 | 83 | 293 | 394 |
| A549-10uM-6h | GSE147507 CL | 68 | 168 | 387 | 2371 | 4596 |
| HA1E-10uM-24h | GSE145926 | 310 | 457 | 846 | 3544 | 3472 |
| HA1E-10uM-24h | GSE147507 PS | 16 | 32 | 83 | 293 | 394 |
| HA1E-10uM-24h | GSE147507 CL | 68 | 168 | 387 | 2371 | 4596 |
| HT29-10uM-24h | GSE145926 | 310 | 457 | 846 | 3544 | 3472 |
| HT29-10uM-24h | GSE147507 PS | 16 | 32 | 83 | 293 | 394 |
| HT29-10uM-24h | GSE147507 CL | 68 | 168 | 387 | 2371 | 4596 |
| MCF7-10uM-24h | GSE145926 | 310 | 457 | 846 | 3544 | 3472 |
| MCF7-10uM-24h | GSE147507 PS | 16 | 32 | 83 | 293 | 394 |
| MCF7-10uM-24h | GSE147507 CL | 68 | 168 | 387 | 2371 | 4596 |
| PC3-10uM-24h | GSE145926 | 310 | 457 | 846 | 3544 | 3472 |
| PC3-10uM-24h | GSE147507 PS | 16 | 32 | 83 | 293 | 394 |
| PC3-10uM-24h | GSE147507 CL | 68 | 168 | 387 | 2371 | 4596 |
| VCAP-10uM-24h | GSE145926 | 310 | 457 | 846 | 3544 | 3472 |
| VCAP-10uM-24h | GSE147507 PS | 16 | 32 | 83 | 293 | 394 |
| VCAP-10uM-24h | GSE147507 CL | 68 | 168 | 387 | 2371 | 4596 |
| VCAP-10uM-6h | GSE145926 | 310 | 457 | 846 | 3544 | 3472 |
| VCAP-10uM-6h | GSE147507 PS | 16 | 32 | 83 | 293 | 394 |
| VCAP-10uM-6h | GSE147507 CL | 68 | 168 | 387 | 2371 | 4596 |

**Table S4.** L1000 genes with expression change at log fold change (LFC) threshold 0.5 were selected to generate severe acute respiratory syndrome coronavirus 2020 (SARS-CoV-2) gene signatures. At this threshold, following connectivity analysis, a large number of candidate drug signatures were identified as discordant to the SARS-CoV-2 signatures (discordance ≤ -0.321). GSE145926: COVID-19 patient samples described in Liao et al.[46] GSE147507 PS: COVID-19 patient samples (lung tissue) described in Blanco-Melo et al[47]. GSE147507 CL: SARS-CoV-2 infected A549_ACE2 cell line sample described in Blanco-Melo et al[47].

**Table S5**

| Drug | Trade name | Drug Class | Target | Canonical MOA | ATC classification | Antiviral properties |
|---|---|---|---|---|---|---|
| **Trametinib** | Mekinist | Antineoplastic; Kinase inhibitor | Mitogen-activated extracellular signal regulated kinase (MEK) 1, 2 | MEK inhibitor | Antineoplastic and immunomodulating agents | MERS-CoV [48], SARS-CoV-2 [49] |
| **Withaferin A** | N/A | Antineoplastic; Steroidal lactone | Unknown | N/A | N/A | SARS-CoV-2 [50-53] |
| **Parthenolide** | N/A | Anti-inflammatory; Sesquiterpene lactone | Unknown | N/A | N/A | SARS [54] |
| **Lapatinib** | Tykerb, Tyverb | Antineoplastic; Kinase inhibitor | Epidermal growth factor receptor; Receptor tyrosine-protein kinase erbB-2 | EGFR, ERBB1 inhibitor | Antineoplastic and immunomodulating agents | SARS-CoV-2 [55] |
| **Sorafenib** | Nexavar | Antineoplastic; Kinase inhibitor | Raf kinase, Platelet-derived growth factor, Vascular endothelial growth factor receptor | Raf/Mek/Erk inhibitor | Antineoplastic and immunomodulating agents | SARS-CoV-2 [56] |
| **Auranofin** | Ridaura | Anti-inflammatory; Gold salt | Peroxiredoxin-5, mitochondrial; Inhibitor of nuclear factor kappa-B kinase subunit beta | Not fully elucidated. | Anti-inflammatory and antirheumatic products | SARS-CoV-2 [57] |
| **Selumetinib** | N/A | Anti-neoplastic; Kinase inhibitor | Mitogen-activated extracellular signal regulated kinase (MEK) 1, 2 | MEK inhibitor | Antineoplastic and immunomodulating agents | SARS-CoV-2[49] |
| **Erlotinib** | Tarceva | Antineoplastic; tyrosine kinase inhibitor | Epidermal growth factor receptor | EGFR receptor | Antineoplastic and immunomodulating agents | HCV, RNA viruses, dengue, Ebola [58-60] |
| **Alvocidib** | N/A | Antineoplastic | Cyclin dependent kinases | Cdk inhibitor | N/A | HSV, HIV, Flu [61-66] |
| **Quinacrine** | Atabrine | Antiparasitic; Antimalarial | DNA | DNA intercalator; full MOA not elucidated | N/A | EMCV, poliovirus |
| **Vandetanib** | Zactima/Zictifa | Antineoplastic; Kinase inhibitor | Vascular endothelial growth factor receptor; Epidermal growth factor | VEGF, EGFR, RET inhibitor | Antineoplastic and immunomodulating agents | Andes virus [67] |

| | | | | | | |
|---|---|---|---|---|---|---|
| | | | receptor; REarranged during Transfection (RET) tyrosine kinases | | | |
| **Dasatinib** | Sprycel | Antineoplastic | Tyrosine protein kinase ABL1; Proto-oncogene tyrosine-protein kinase Src; Ephrin type-A receptor 2; Tyrosine-protein kinase Lck | BCR/ABL and Src family tyrosine kinase inhibitor | Antineoplastic and immunomodulating agents | HIV [68,69] |
| **Thioridazine** | Mellaril/Melleril | Antipsychotic; Phenothiazine | Dopamine D1/D2 receptors, Alpha-1A/1B adrenergic receptor | Inhibition of mesolimbic dopaminergic D1 and D2 receptors | Psycholeptics | Ebola [70,71], HCV [72]; CHIKV, SFV [73]; RVFV [74] |
| **Candidate repurposable drugs currently in trial for COVID-19** | | | | | | |
| **Gallocatechin Gallate** | N/A (Veregen, Sinecatechin) | Antioxidant | Unknown | Not fully elucidated | Antibiotics and chemotherapeutics for dermatological use (as a Sinecatechin) | |
| **Decitabine** | Dacogen | Antineoplastic; cytosine analogue | DNA, nucleotide analog | Leads to DNA hypomethylation by trapping DNA methyltransferases | Antineoplastic and immunomodulating agents | |
| **Fenretinide** | N/A | Antineoplastic; chemopreventive synthetic retinoid | Not fully elucidated | Inhibits cell growth through retinoid receptor dependent and independent pathways | N/A | |
| **Curcumin** | N/A | Anti-inflammatory, antimicrobial, antioxidant | Not fully elucidated | Antioxidant, reactive oxygen species scavenger | N/A | |
| **Simvastatin** | Zocor | Antilipemic | 3-hydroxy-3-methylglutaryl-coenzyme A (HMG-CoA) reductase | HMG-CoA reductase inhibitor | Lipid modifying agent | |

| | | | | | | |
|---|---|---|---|---|---|---|
| **Sirolimus** | Rapamune | Immuno-suppressant; Macrolide lactams | Serine/threonine-protein kinase mTOR | mTOR inhibitor | Immunosuppressants | |
| **Cyclosporine** | Cequa | Immuno-suppressant | Calcium signal-modulating cyclophilin ligand | Calcineurin inhibitor | N/A | |

**Table S5.**

Detailed information on the top 20 candidate drugs. Information relating to these drugs (were referenced from DrugBank (https://www.drugbank.ca/) and Anatomical Therapeutic Chemical (ATC) classification (https://www.whocc.no/atc_ddd_index/) databases. The first-level of ATC classification is indicated.

**Figure S1**

**Figure S1**

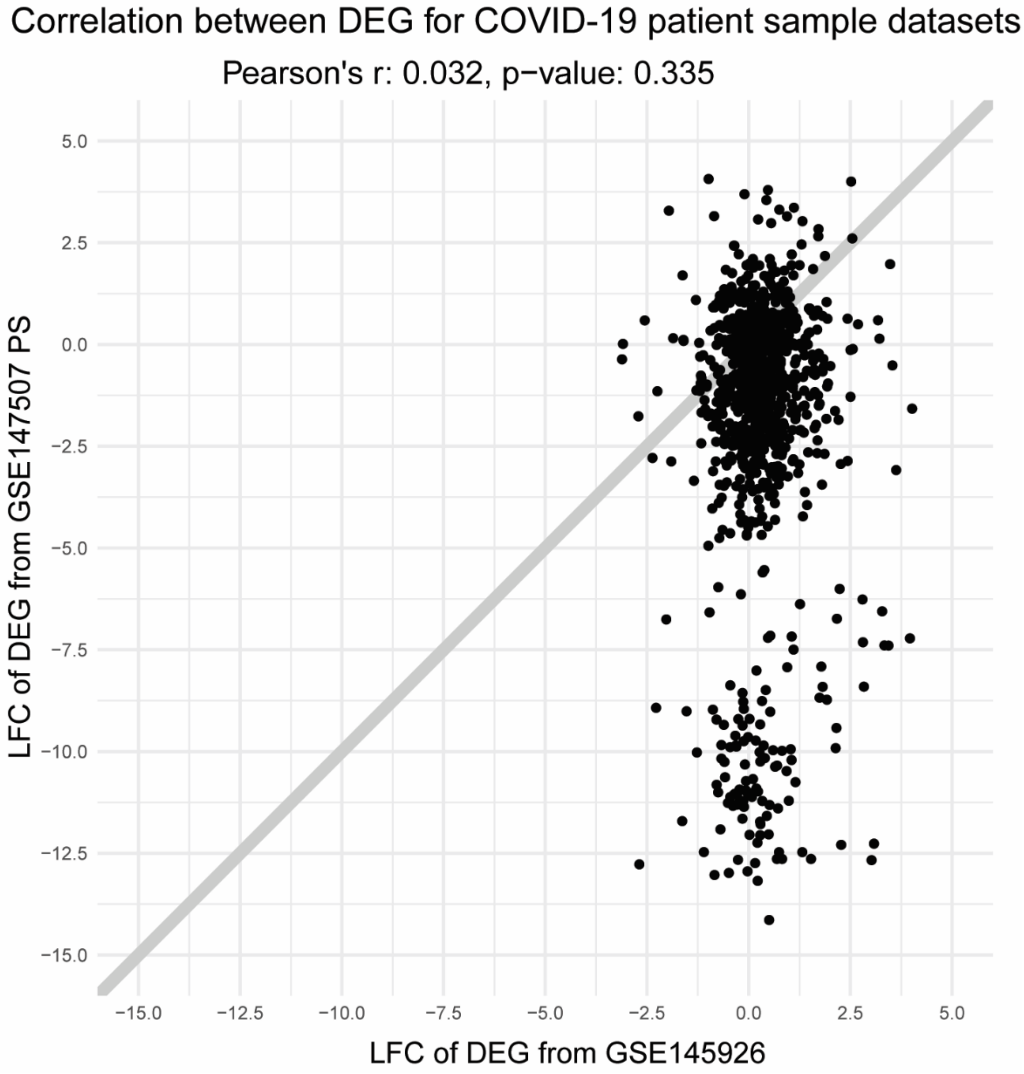


**Figure S1.** Correlation between COVID-19 patient sample datasets GSE147507 PS and GSE145926. There was no significant correlation between the log fold change (LFC) differential gene expression (DEG) values for GSE147507 PS[24] and GSE145926[25] COVID-19 patient samples, derived from lung tissue and bronchoalveolar fluid immune cells, respectively.

## Figure S2

A

| GSE147507 CL | | |
|---|---|---|
| **Pathway name** | **# Genes found** | **FDR** |
| **Signaling by Interleukins** | 64 | 6.30E-09 |
| **Interleukin-4 and Interleukin-13 signaling** | 32 | 9.16E-08 |
| Cytokine Signaling in Immune system | 85 | 2.17E-07 |
| **Signaling by Receptor Tyrosine Kinases** | 57 | 6.31E-07 |
| Generic Transcription Pathway | 96 | 3.09E-04 |
| Extra-nuclear estrogen signaling | 17 | 4.02E-04 |
| Estrogen-dependent nuclear events downstream of ESR-membrane signaling | 9 | 4.22E-04 |
| Response of EIF2AK1 (HRI) to heme deficiency | 9 | 4.22E-04 |
| MyD88-independent TLR4 cascade | 16 | 7.32E-04 |
| TRIF(TICAM1)-mediated TLR4 signaling | 16 | 7.32E-04 |
| Immune System | 144 | 9.37E-04 |
| ESR-mediated signaling | 26 | 9.37E-04 |
| Signaling by NTRKs | 20 | 9.37E-04 |
| TRAF6 mediated induction of NFkB and MAP kinases upon TLR7/8 or 9 activation | 15 | 9.54E-04 |
| Toll Like Receptor 3 (TLR3) Cascade | 15 | 9.54E-04 |

B

| GSE147507 PS | | |
|---|---|---|
| **Pathway name** | **# Genes found** | **FDR** |
| Mitotic G1 phase and G1/S transition | 49 | 5.97E-13 |
| Cell Cycle | 107 | 3.25E-10 |
| Cell Cycle, Mitotic | 90 | 3.06E-09 |
| **Signaling by Interleukins** | 94 | 5.93E-09 |
| G1/S Transition | 37 | 2.14E-08 |
| Transcriptional Regulation by TP53 | 75 | 3.87E-08 |
| **Signaling by Receptor Tyrosine Kinases** | 89 | 4.56E-08 |
| **Interleukin-4 and Interleukin-13 signaling** | 44 | 8.43E-08 |
| Apoptosis | 39 | 5.28E-07 |
| S Phase | 37 | 1.33E-06 |
| Signaling by NTRKs | 35 | 1.65E-06 |
| Cell Cycle Checkpoints | 48 | 2.05E-06 |
| Cyclin A:Cdk2-associated events at S phase entry | 24 | 4.50E-06 |
| Programmed Cell Death | 40 | 4.92E-06 |
| Gene expression (Transcription) | 186 | 6.07E-06 |

C

| GSE145926 | | |
|---|---|---|
| **Pathway name** | **# Genes found** | **FDR** |
| Mitotic G1 phase and G1/S transition | 32 | 2.26E-09 |
| **Signaling by Receptor Tyrosine Kinases** | 66 | 2.26E-09 |
| **Interleukin-4 and Interleukin-13 signaling** | 33 | 7.41E-08 |
| **Signaling by Interleukins** | 63 | 7.41E-08 |
| Cell Cycle | 67 | 2.10E-07 |
| G0 and Early G1 | 14 | 2.41E-07 |
| Generic Transcription Pathway | 111 | 4.82E-07 |
| Cell Cycle, Mitotic | 57 | 5.43E-07 |
| TP53 Regulates Transcription of Cell Cycle Genes | 16 | 2.94E-06 |
| G1/S Transition | 24 | 3.11E-06 |
| TP53 Regulates Transcription of Genes Involved in G2 Cell Cycle Arrest | 10 | 3.11E-06 |
| Estrogen-dependent nuclear events downstream of ESR-membrane signaling | 11 | 5.37E-06 |
| Transcriptional regulation by the AP-2 (TFAP2) family of transcription factors | 14 | 5.37E-06 |
| Transcriptional Regulation by TP53 | 47 | 5.93E-06 |
| RNA Polymerase II Transcription | 113 | 6.06E-06 |

D

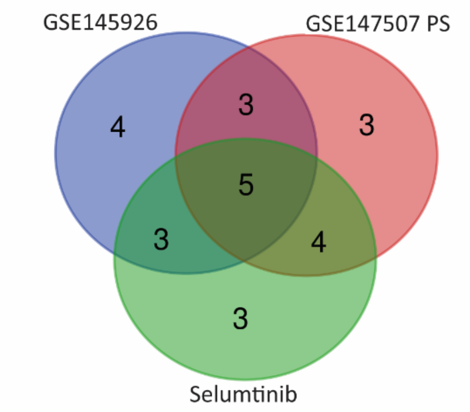


E

| Selumtinib | | |
|---|---|---|
| **Pathway name** | **# Genes found** | **FDR** |
| Mitotic G1 phase and G1/S transition | 38 | 5.31E-14 |
| Cell Cycle, Mitotic | 72 | 5.31E-14 |
| Cell Cycle | 84 | 5.31E-14 |
| G1/S Transition | 31 | 2.66E-11 |
| Transcriptional Regulation by TP53 | 56 | 1.23E-10 |
| S Phase | 32 | 3.60E-10 |
| G0 and Early G1 | 16 | 7.46E-10 |
| Cell Cycle Checkpoints | 39 | 1.52E-09 |
| Gene expression (Transcription) | 125 | 1.68E-08 |
| Generic Transcription Pathway | 110 | 1.93E-08 |
| RNA Polymerase II Transcription | 114 | 1.38E-07 |
| Cyclin E associated events during G1/S transition | 19 | 3.03E-07 |
| Cyclin A:Cdk2-associated events at S phase entry | 19 | 4.00E-07 |
| Resolution of AP sites via the multiple-nucleotide patch replacement pathway | 12 | 6.56E-07 |
| DNA Replication | 23 | 1.11E-06 |

F

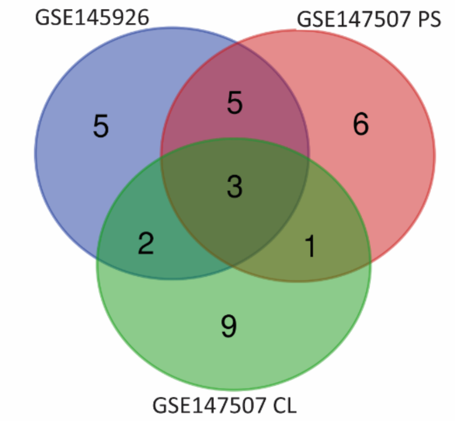


**Figure S2. Biological pathway analysis of datasets** GSE147507 CL (A), GSE147507 PS (B), and GSE145926 (C). Venn diagram shows common (in bold) and unique pathways (D). Biological pathway analysis of the candidate drug selumetinib (E) and venn diagram (F) showing common pathways (red) between COVID-19 patient sample datasets (GSE147507 PS and GSE145926) and selumetinib.

**References**


1. Drugbank. Hydroxychloroquine. (2007).
2. Wang, M., *et al.* Remdesivir and chloroquine effectively inhibit the recently emerged novel coronavirus (2019-nCoV) in vitro. *Cell research* **30**, 269-271 (2020).
3. Gao, J., Tian, Z., Yang, X. Breakthrough: Chloroquine Phosphate Has Shown Apparent Efficacy in Treatment of COVID-19 Associated Pneumonia in Clinical Studies. *Biosci Trends* **14**, 72-73 (2020).
4. Gautret, P., Lagier, J.C., Parola, P., Hoang, V.T., Meddeb, L., Mailhe, M., Doudier, B., Courjon, J., Giordanengo, V., Vieira, V.E., Dupont, T., Honoré, S., Colson, P., Chabriere, E., La Scola, B., Rolain, J.M., Brouqui, P., Raoult, D. Hydroxychloroquine and azithromycin as a treatment of COVID-19: results of an open-label non-randomized clinical trial. *Int. J. Antimicrob. Agents* (2020).
5. Colson, P., Rolain, J.M., Lagier, J.C., Brouqui, P. & Raoult, D. Chloroquine and hydroxychloroquine as available weapons to fight COVID-19. *International journal of antimicrobial agents*, 105932 (2020).
6. Rolain, J.M., Colson, P. & Raoult, D. Recycling of chloroquine and its hydroxyl analogue to face bacterial, fungal and viral infections in the 21st century. *International journal of antimicrobial agents* **30**, 297-308 (2007).
7. Drugbank. Chloroquine. (2005).
8. Drugbank. Lopinavir. (2007).
9. Yao, T.T., Qian, J.D., Zhu, W.Y., Wang, Y. & Wang, G.Q. A systematic review of lopinavir therapy for SARS coronavirus and MERS coronavirus-A possible reference for coronavirus disease-19 treatment option. *J Med Virol* (2020).
10. Deng, L., *et al.* Arbidol combined with LPV/r versus LPV/r alone against Corona Virus Disease 2019:a retrospective cohort study. *The Journal of infection* (2020).
11. Cao, B., *et al.* A Trial of Lopinavir-Ritonavir in Adults Hospitalized with Severe Covid-19. *N Engl J Med* (2020).
12. Martinez, M.A. Compounds with therapeutic potential against novel respiratory 2019 coronavirus. *Antimicrobial agents and chemotherapy* (2020).
13. Hurst, M. & Faulds, D. Lopinavir. *Drugs* **60**, 1371-1379; discussion 1380-1371 (2000).
14. Drugbank. Ritonavir. (2005).
15. Drugbank. Fedratinib. (2016).
16. Stebbing, J., *et al.* COVID-19: combining antiviral and anti-inflammatory treatments. *Lancet Infect Dis* (2020).
17. Wu, D., Yang, X.O. TH17 responses in cytokine storm of COVID-19: An emerging target of JAK2inhibitor Fedratinib. *J. Microbiol. Immunol.* (2020).
18. Drugbank. Ruxolitinib. (2013).
19. Drugbank. Baricitinib. (2016).
20. Drugbank. Azithromycin. (2005).
21. Drugbank. Losartan. (2005).
22. Gurwitz, D. Angiotensin receptor blockers as tentative SARS-CoV-2 therapeutics. *Drug Dev Res* (2020).
23. Drugbank. Remdesivir. (2019).

24. Sheahan, T.P., *et al.* Broad-spectrum antiviral GS-5734 inhibits both epidemic and zoonotic coronaviruses. *Sci Transl Med* **9**(2017).
25. Agostini, M.L., *et al.* Coronavirus Susceptibility to the Antiviral Remdesivir (GS-5734) Is Mediated by the Viral Polymerase and the Proofreading Exoribonuclease. *mBio* **9**(2018).
26. de Wit, E., *et al.* Prophylactic and therapeutic remdesivir (GS-5734) treatment in the rhesus macaque model of MERS-CoV infection. *Proc Natl Acad Sci U S A* (2020).
27. Ko, W., Rolain, J.M., Lee, N.Y., Chen, P.L., Huang, C.T., Lee, P.I., Hsueh, P.R. Arguments in Favour of Remdesivir for Treating SARS-CoV-2 Infections. *Int. J. Antimicrob. Agents* (2020).
28. Muller, C., *et al.* Comparison of broad-spectrum antiviral activities of the synthetic rocaglate CR-31-B (-) and the eIF4A-inhibitor Silvestrol. *Antiviral research* **175**, 104706 (2020).
29. Muller, C., *et al.* Broad-spectrum antiviral activity of the eIF4A inhibitor silvestrol against corona- and picornaviruses. *Antiviral research* **150**, 123-129 (2018).
30. Drugbank. Umifenovir. (2017).
31. Wang, Z., Chen, X., Lu, Y., Chen, F. & Zhang, W. Clinical characteristics and therapeutic procedure for four cases with 2019 novel coronavirus pneumonia receiving combined Chinese and Western medicine treatment. *Biosci Trends* **14**, 64-68 (2020).
32. Lu, H. Drug treatment options for the 2019-new coronavirus (2019-nCoV). *Biosci Trends* **14**, 69-71 (2020).
33. Deng, L., *et al.* Arbidol combined with LPV/r versus LPV/r alone against Corona Virus Disease 2019: A retrospective cohort study. *The Journal of infection* (2020).
34. Smee, D.F., *et al.* Novel pyrazolo[3,4-d]pyrimidine nucleoside analog with broad-spectrum antiviral activity. *Antimicrobial agents and chemotherapy* **31**, 1535-1541 (1987).
35. Drugbank. Interferon Alphacon 1. (2005).
36. Li, G. & De Clercq, E. Therapeutic options for the 2019 novel coronavirus (2019-nCoV). *Nature reviews. Drug discovery* **19**, 149-150 (2020).
37. Du, B., *et al.* [Pharmacotherapeutics for the new coronavirus pneumonia]. *Zhonghua jie he he hu xi za zhi = Zhonghua jiehe he huxi zazhi = Chinese journal of tuberculosis and respiratory diseases* **43**, 173-176 (2020).
38. Luo, W., *et al.* Anti-SARS coronavirus 3C-like protease effects of Rheum palmatum L. extracts. *Biosci Trends* **3**, 124-126 (2009).
39. Ho, T.Y., Wu, S.L., Chen, J.C., Li, C.C. & Hsiang, C.Y. Emodin blocks the SARS coronavirus spike protein and angiotensin-converting enzyme 2 interaction. *Antiviral research* **74**, 92-101 (2007).
40. Drugbank. Camostat. (2017).
41. Yamawaki, H., *et al.* Camostat Mesilate, Pancrelipase, and Rabeprazole Combination Therapy Improves Epigastric Pain in Early Chronic Pancreatitis and Functional Dyspepsia with Pancreatic Enzyme Abnormalities. *Digestion* **99**, 283-292 (2019).
42. Ramsey, M.L., Nuttall, J., Hart, P.A. & Team, T.I. A phase 1/2 trial to evaluate the pharmacokinetics, safety, and efficacy of NI-03 in patients with chronic pancreatitis: study protocol for a randomized controlled trial on the assessment of camostat treatment in chronic pancreatitis (TACTIC). *Trials* **20**, 501 (2019).

43. Zhou, Y., *et al.* Protease inhibitors targeting coronavirus and filovirus entry. *Antiviral research* **116**, 76-84 (2015).
44. Shirato, K., Kawase, M. & Matsuyama, S. Middle East respiratory syndrome coronavirus infection mediated by the transmembrane serine protease TMPRSS2. *Journal of virology* **87**, 12552-12561 (2013).
45. Kawase, M., Shirato, K., van der Hoek, L., Taguchi, F. & Matsuyama, S. Simultaneous treatment of human bronchial epithelial cells with serine and cysteine protease inhibitors prevents severe acute respiratory syndrome coronavirus entry. *Journal of virology* **86**, 6537-6545 (2012).
46. Liao, M., *et al.* Single-cell landscape of bronchoalveolar immune cells in patients with COVID-19. *Nature medicine* **26**, 842-844 (2020).
47. Blanco-Melo, D., *et al.* Imbalanced Host Response to SARS-CoV-2 Drives Development of COVID-19. *Cell* **181**, 1036-1045 e1039 (2020).
48. Kindrachuk, J., *et al.* Antiviral potential of ERK/MAPK and PI3K/AKT/mTOR signaling modulation for Middle East respiratory syndrome coronavirus infection as identified by temporal kinome analysis. *Antimicrobial agents and chemotherapy* **59**, 1088-1099 (2015).
49. Zhou, L., *et al.* MEK inhibitors reduce cellular expression of ACE2, pERK, pRb while stimulating NK-mediated cytotoxicity and attenuating inflammatory cytokines relevant to SARS-CoV-2 infection. *Oncotarget* **11**, 4201-4223 (2020).
50. Tripathi, M.K., *et al.* Identification of bioactive molecule from Withania somnifera (Ashwagandha) as SARS-CoV-2 main protease inhibitor. *Journal of biomolecular structure & dynamics*, 1-14 (2020).
51. Kumar, V., *et al.* Withanone and Withaferin-A are predicted to interact with transmembrane protease serine 2 (TMPRSS2) and block entry of SARS-CoV-2 into cells. *Journal of biomolecular structure & dynamics*, 1-13 (2020).
52. Kumar, V., Dhanjal, J.K., Kaul, S.C., Wadhwa, R. & Sundar, D. Withanone and caffeic acid phenethyl ester are predicted to interact with main protease (M(pro)) of SARS-CoV-2 and inhibit its activity. *Journal of biomolecular structure & dynamics*, 1-13 (2020).
53. Straughn, A.R. & Kakar, S.S. Withaferin A: a potential therapeutic agent against COVID-19 infection. *J Ovarian Res* **13**, 79 (2020).
54. DeDiego, M.L., *et al.* Inhibition of NF-kappaB-mediated inflammation in severe acute respiratory syndrome coronavirus-infected mice increases survival. *Journal of virology* **88**, 913-924 (2014).
55. Raymonda, M.H., *et al.* Pharmacologic profiling reveals lapatinib as a novel antiviral against SARS-CoV-2 in vitro. *BioRxiv* **preprint**(2020).
56. Ellinger, B., *et al.* Identification of inhibitors of SARS-CoV-2 in-vitro cellular toxicity in human (Caco-2) cells using a large scale drug repurposing collection. *Research Square* **preprint**(2020).
57. Rothan, H.A., *et al.* The FDA-approved gold drug auranofin inhibits novel coronavirus (SARS-COV-2) replication and attenuates inflammation in human cells. *Virology* **547**, 7-11 (2020).

58. Bekerman, E., *et al.* Anticancer kinase inhibitors impair intracellular viral trafficking and exert broad-spectrum antiviral effects. *The Journal of clinical investigation* **127**, 1338-1352 (2017).
59. Pu, S.Y., *et al.* Feasibility and biological rationale of repurposing sunitinib and erlotinib for dengue treatment. *Antiviral research* **155**, 67-75 (2018).
60. Stebbing, J., *et al.* COVID-19: combining antiviral and anti-inflammatory treatments. *Lancet Infect Dis* **20**, 400-402 (2020).
61. Yamamoto, M., *et al.* CDK9 inhibitor FIT-039 prevents replication of multiple DNA viruses. *J Clin Invest* **124**, 3479-3488 (2014).
62. Schang, L.M., *et al.* Pharmacological cyclin-dependent kinase inhibitors inhibit replication of wild-type and drug-resistant strains of herpes simplex virus and human immunodeficiency virus type 1 by targeting cellular, not viral, proteins. *J Virol* **76**, 7874-7882 (2002).
63. Diwan, P., Lacasse, J.J. & Schang, L.M. Roscovitine inhibits activation of promoters in herpes simplex virus type 1 genomes independently of promoter-specific factors. *J Virol* **78**, 9352-9365 (2004).
64. Biglione, S., *et al.* Inhibition of HIV-1 replication by P-TEFb inhibitors DRB, seliciclib and flavopiridol correlates with release of free P-TEFb from the large, inactive form of the complex. *Retrovirology* **4**, 47 (2007).
65. Zhou, M., *et al.* Coordination of transcription factor phosphorylation and histone methylation by the P-TEFb kinase during human immunodeficiency virus type 1 transcription. *J Virol* **78**, 13522-13533 (2004).
66. Wang, S., Zhang, J. & Ye, X. [Protein kinase inhibitor flavopiridol inhibits the replication of influenza virus in vitro]. *Wei Sheng Wu Xue Bao* **52**, 1137-1142 (2012).
67. Gasparian, A.V., *et al.* Inhibition of encephalomyocarditis virus and poliovirus replication by quinacrine: implications for the design and discovery of novel antiviral drugs. *Journal of virology* **84**, 9390-9397 (2010).
68. Bermejo, M., *et al.* Dasatinib inhibits HIV-1 replication through the interference of SAMHD1 phosphorylation in CD4+ T cells. *Biochem Pharmacol* **106**, 30-45 (2016).
69. Salgado, M., *et al.* Dasatinib protects humanized mice from acute HIV-1 infection. *Biochem Pharmacol* **174**, 113625 (2020).
70. Johansen, L.M., *et al.* A screen of approved drugs and molecular probes identifies therapeutics with anti-Ebola virus activity. *Sci Transl Med* **7**, 290ra289 (2015).
71. Lee, N., *et al.* High-throughput drug screening using the Ebola virus transcription- and replication-competent virus-like particle system. *Antiviral research* **158**, 226-237 (2018).
72. Chamoun-Emanuelli, A.M., *et al.* Phenothiazines inhibit hepatitis C virus entry, likely by increasing the fluidity of cholesterol-rich membranes. *Antimicrobial agents and chemotherapy* **57**, 2571-2581 (2013).
73. Pohjala, L., *et al.* Inhibitors of alphavirus entry and replication identified with a stable Chikungunya replicon cell line and virus-based assays. *PloS one* **6**, e28923 (2011).
74. Filone, C.M., *et al.* Rift valley fever virus infection of human cells and insect hosts is promoted by protein kinase C epsilon. *PloS one* **5**, e15483 (2010).
